# Assessment of a demonstrator repository for individual clinical trial data built upon DSpace

**DOI:** 10.12688/f1000research.23468.2

**Published:** 2020-06-25

**Authors:** Birol Tilki, Thomas Schulenberg, Steve Canham, Rita Banzi, Wolfgang Kuchinke, Christian Ohmann

**Affiliations:** 1 Coordination Centre for Clinical Trials, Heinrich-Heine-University, Düsseldorf, Nordrhine-Westfalia, 40225, Germany; 2 European Clinical Research Infrastructure Network, ECRIN, Redhill, Surrey, RH1 6QH, UK; 3 Istituto di Ricerche Farmacologiche Mario Negri, IRCCS, Milan, 20156, Italy; 4 European Clinical Research Infrastructure Network, ECRIN, Düsseldorf, Nordrhine-Westfalia, 40477, Germany

**Keywords:** Repository, clinical trial, individual participant data, data sharing, DSpace

## Abstract

**Background:** Given the increasing number and heterogeneity of data repositories, an improvement and harmonisation of practice within repositories for clinical trial data is urgently needed. The objective of the study was to develop and evaluate a demonstrator repository, using a widely used repository system (DSpace), and then explore its suitability for providing access to individual participant data (IPD) from clinical research.

**Methods:** After a study of the available options, DSpace (version 6.3) was selected as the software for developing a demonstrator implementation of a repository for clinical trial data. In total, 19 quality criteria were defined, using previous work assessing clinical data repositories as a guide, and the demonstrator implementation was then assessed with respect to those criteria.

**Results:** Generally, the performance of the DSpace demonstrator repository in supporting sensitive personal data such as that from clinical trials was strong, with 14 requirements demonstrated (74%), including the necessary support for metadata and identifiers. Two requirements could not be demonstrated (the ability to include de-identification tools and the availabiltiy of a self-attestation system) and three requirements were only partially demonstrated (ability to provide links to de-identification tools and requirements, incorporation of a data transfer agreement in system workflow, and capability to offer managed access through application on a case by case basis).

**Conclusions:** Technically, the system was able to support most of the pre-defined requirements, though there are areas where support could be improved. Of course, in a productive repository, appropriate policies and procedures would be needed to direct the use of the available technical features. A technical evaluation should therefore be seen as indicating a system’s potential, rather than being a definite assessment of its suitability. DSpace clearly has considerable potential in this context and appears a suitable base for further exploration of the issues around storing sensitive data.

## Introduction

The sharing of clinical trial data still occurs mainly with in a closed professional evironment through direct and personal sharing, rather than via accessible data repositories. A multi-stakeholder taskforce addressing this problem recommended that data and documents from clinical trials available for sharing should be transferred to a suitable data repository to help ensure that the data objects are properly prepared, are available in the longer term, are stored securely and are subject to rigorous governance ^1^. A recent study has shown that an increasing number of such repositories are available for sharing of individual participant data (IPD) from clinical studies ^2^. There are many different types of repositories, however, such as generic repositories for all kinds of life-science data, repositories exclusively for clinical research data and specialised repositories with a specific focus, e.g. a single disease area, and major heterogeneity exists with respect to data-upload, data-handling, and data-access processes. This heterogeneity of repository types and features, reflects both the different purposes and perspectives of repository founders, and the relative immaturity of repository data-sharing services. Given the lack of a consensus about the services required from a data repository, each organisation has implemented its own policies and systems to meet its own priorities. Greater harmonisation of practices within repositories, coupled with the implementation of quality criteria for repositories, may diminish the reluctance of many researchers to share the data from their studies, thus promoting data-sharing, discoverability, and re-use ^3, 4^.

In a consensus building exercise, the necessity for compliance of repositories for clinical trial data and related data objects with quality criteria was emphasised ^1^. The services any repository provides should conform to specified quality standards, to give its users confidence that their data and documents will be stored securely and in accordance with the specific data transfer agreements they have agreed. During the consensus exercise, the importance of getting consent for data archiving, sharing and re-use from research participants was stressed and formulated as one of the essential data sharing principles.

This paper explores the suitability of a widely used data repository system, DSpace, for supporting the long-term management of IPD generated from clinical research while conforming to defined quality criteria. Though DSpace is a repository system used for open data, it is increasingly used also for restricted data access because it provides several built-in features that make it adaptable for restricted data sharing. The work was carried out as part of a broader set of activities aimed at developing mechanisms for the sharing of IPD from clinical research ( https://www.corbel-project.eu/home.htm). It builds on previous published papers describing principles and practical recommendations for IPD sharing ^1^, offering a detailed analysis of the processes involved in depositing, managing and sharing IPD ^5^, and evaluating existing repositories for their suitability for the deposition of IPD, specifically for researchers in the non-commercial sector ^2^. In the latter analysis, repositories were assessed against a set of quality criteria, referring to the processes of data upload, storage, de-identification, and quality controls, metadata, identifiers, flexibility of access and long-term preservation. The aim of this paper is to describe the development of a demonstrator repository based on the DSpace system and assess it using a pre-defined set of quality criteria and requirements.

The reason for developing this repository was to explore further various technical and workflow issues around the long-term management of IPD, in practical terms, using a well-known repository system applied to IPD from clinical research. The demonstrator is intended as an illustrative example only and this paper deals only with technical aspects of the repository system, i.e. its evaluation as a suitable infrastructure. It is clear that many aspects of a repository’s suitability for IPD are linked to the procedures and processes implemented by the institution hosting the repository. In other words, a strong technical infrastructure is a necessary but not sufficient indicator of quality.

## Methods

### Selection of DSpace as software for developing a demonstrator repository

Writing a bespoke repository system from scratch was seen as unrealistic, given resource constraints, and in any case less useful than using an existing system – one that would also be available to potential repository managers. A variety of systems were considered as the possible base system for the demonstrator repository (e.g. Figshare, DSpace). These and other systems were characterised with respect to the following standardised criteria ^6^:

- Name of the system
- Contact
- Webpage of the system
- Level of usage (country)
- Short description of the system
- Type of activity the system is supporting
- Modules/architecture/components included
- What data stored with the system
- Research use cases/projects/studies the system is used

A formal comparison between the systems was not made ^6^, but DSpace was rated as the system with the greatest potential for a demonstrator repository, particularly in an academic context.

A formal comparison between the systems was not made ^6^, but DSpace was rated as the system with the greatest potential for a demonstrator repository, particularly in an academic context.

DSpace was selected partly because it appears to be by far the most popular of the various repository systems, with almost 2884 users, 2204 of them listed as ‘academic’ (including the University of Cambridge, Yale, Duke University and the University of Edinburgh amongst many around the world; https://duraspace.org/registry/). Three of 25 repositories for IPD from clinical trial data, evaluated in a recent review, are built upon DSpace (Dryad, Drum, Edinburgh DataShare) ^2^.

In addition, DSpace is an open source system and can be modified and extended by users. It claims about 100 contributors to the code base, with the Dryad repository, which runs on DSpace, being an example of how the system can be extended. It is possible to download and run a pre-configured ‘out of the box’ solution, but DSpace also claims to be fully modifiable, even though many of the modifications listed are relatively superficial (e.g. themes, screen configurations, search parameters). The system appeares compliant with most of the relevant standards (e.g. Open Archives Initiative Protocol for Metadata (OAI-PMH)), developed for harvesting metadata descriptions from records), runs on a variety of operating systems and can use either Oracle or PostgreSQL as the back-end database store ( https://duraspace.org/dspace/). There also appeared to be an active user group and comprehensive documentation, including a Wiki ( https://wiki.duraspace.org/display/DSPACE/). An alternative to DSpace would have been Invenio ( https://invenio-software.org/), which delivers the repository units for Zenodo, OpenAIRE and CERN Open Data. Invenio appeared very focused on open data, however, while DSpace seemed to offer more possibilities for supporting more managed access. Further details of the candidate systems considered are given in 6.

### Technical infrastructure for the demonstrator repository

A data repository was established between October 2018 and June 2019 within the Coordination Centre for Clinical Trials at the University of Düsseldorf, by BT (first author) using version 6.3 of DSpace. Additional software was installed to supplement DSpace functioning and manage servers and common server functionality.

*Full list of the software and hardware used for the repository installations and details of the technical implementation of the demonstrator repository:*

DSpace is a framework of a considerable number of different software tools that must work together to achieve an efficient DSpace installation. Prerequisite software tools must be downloaded, installed, tested, configured and integrated with each other. In addition to DSpace itself, the following were installed:

- Ubuntu 16 and Ubuntu 18 (Linux operating system)
- Java 8 (Java Development Kit)
- Apache Maven 3.3.9 (Java build tool)
- Apache Ant 1.9.13 (Java build tool)
- PostgreSQL 9.5 (with pgcrypto installed) as the relational database back end
- Apache Tomcat 9.0.11 (Java Servlet, Server Pages, and Web Socket Engine)

DSpace can be installed at different scales, allowing different amounts of data to be handled. In our usage scenario we assumed the storage of several hundred trials with a size of 10–100 MB per trial, uploaded over several years. We therefore decided to install a mid-range version of DSpace, able to accommodate a large number of clinical trials datasets. The virtual server was established with:

- 6 GB RAM in total: approximately 2 GB for Ubuntu 16/18, 2 GB for PostgreSQL, 2 GB for Tomcat.
- 200 GB system storage. Deducting 40 GB for system and application use this provides enough storage for 1600 datasets (at 100MB per dataset).

This mid-range system is capable of supporting an application with either a large number of items (roughly 50,000 files and associated metadata) or a large volume of activity (searches, accesses, downloads, etc.).

For testing, publicly available data and documents as from clinical trials were uploaded to the demonstrator repository. The data used are displayed on the welcome page of the DSpace demonstrator repository ( http://90.147.75.211:8080/xmlui/).

### Quality applied to the reference implementation

The quality criteria used for assessment were developed from an original collection of 34 attributes, themselves derived from previous work and discussion within CORBEL and the IMPACT Observatory project ^2^. These criteria were meant to provide a broad characterisation of a repository and included aspects assessing both a repository’s relative maturity and its suitability for clinical research data. From these criteria 8 features were selected as being especially important for clinical researchers wishing to deposit individual participant data (IPD). They were used in a general evaluation of repositories ^2^ and were also applied to the DSpace implementation.

These 8 criteria identified as being key to successful management of IPD are listed below ^2^.

1. Guidelines for data upload and storage
2. Support for data de-identification
3. Data quality controls
4. Contracts for upload and storage
5. Available provenance and accessibility metadata
6. Application of identifiers
7. Flexibility of access
8. Repository long term preservation

Other standards and criteria for trustworthy digital repositories have been developed and are being applied, e.g., Data Seal of Approval, International Council for Science World Data Systems ^7– 10^). These criteria usually examine more generic repository features, for example the nature of the security measures in place, the use of encryption, the technical infrastructure, staff competence, etc. Because in this exercise we were not evaluating a repository, but focusing instead on a specific tool, one that would sit *within* a repository, we did not look at these more general criteria in detail. Of course activities such as monitoring, reviewing and implementing security measures are very important, but we would see them mainly as the concern of the repository managing DSpace rather than DSpace itself. The relationship between the eight criteria used here and other standards and criteria available for repositories is explored further in the Discussion section (see also Table 3).

Managing metadata (data about data) is a key requirement of any repository system, though there are two distinct forms of metadata to consider. To promote interoperability and retain meaning within interpretation and analysis, shared data should be, as far as possible, structured, described and formatted using widely recognised data and metadata standards (e.g. Clinical Data Interchange Standards Consortium (CDISC), Core Outcome Measures in Effectiveness Trials (COMET), Medical Dictionary for Regulatory Activities (MedDRA)) ^1^. The metadata in this context is *descriptive*, detailing the contents of the data. A repository should be able to check that such metadata is available, ideally in one of a range of specified formats, and support its inclusion with the data (see the details for criteria 1) but the responsibility for providing it rests with the data generators. But there is also a need for *provenance and accessibility* metadata, which is used to make up a repository's catalogue of content, and which describes, for example, the nature and source of the data, its date(s), the authors, and – especially important with sensitive data that is likely to be under managed access – how the data can be accessed, including the details of any application procedure. Providing such metadata is the responsibility of the repository itself, although ideally it is done in close collaboration with the data generators. This type of metadata is the subject of criterion 5.

In order to make the assessment of the criteria more operational and to distinguish features of the system (technical features) from measures around the system (e.g. policies and procedures), the criteria were split into specific requirements. This was performed by the group of authors. Table 1 provides a detailed breakdown of the eight criteria in terms of their associated ‘requirements’ – i.e. the features one would normally expect to see implemented. ‘System’ features (i.e. repository system and its technical features), are distinguished from ‘Procedures’ (i.e. function of the repository’s policies and procedures).

**Table 1.** Quality criteria and linked requirements. *System:* To be demonstrated by the repository system’s technical features. *Procedures:* Function of the repository’s governance, policies, procedures.

Requirement	
1. Guidelines for data upload and storage (The repository should …)	
1a. support a range of file types and metadata schema	System
1b. provide mechanisms for the upload of files, including instructions	System
1c. provide rules and guidelines for data upload and storage	Procedures
2. De-identification practices before upload (The repository should …)	
2a. be able to provide links to de-identification tools and requirements	System
2b. implement de-identification tools	System
2c. provide requirements and / or guidelines for de-identification	Procedures
2d. provide a consultancy service on de-identification	Procedures
3. Control of quality of data (The repository should …)	
3a. support quality control in its workflow	System
3b. enforce procedures that promote and monitor data and metadata quality	Procedures
4. Formal contract regarding upload and storage (The repository should …)	
4a. incorporate a data transfer agreement in system workflow	System
4b. make a comprehensive data transfer agreement mandatory	Procedures
5. Application of a metadata schema to describe contents (The repository should …)	
5a. use a consistent metadata schema to describe its content	System
5b. allow a customised metadata schema to be applied	System
5c. provide tools to help data generators to complete metadata fields	System
5d. make metadata openly (public) available	System
5e. have policies in place that enforce the application of appropriate metadata	Procedures
6. Application of an identifier (The repository should …)	
6a. be able to apply a primary persistent identifier system	System
6b. be able to use other persistent identifiers as appropriate	System
6c. have policies and processes that ensure identifiers are applied correctly	Procedures
7. Flexibility of access (The repository should …)	
7a. allow open access to material, with an optional embargo period	System
7b. allow open access after web-based self-attestation of the user	System
7c. offer managed access through group membership	System
7d. offer managed access through application on a case by case basis	System
7e. support granular access to different parts of datasets collections	System
7f. have policies that ensure access is specified and monitored	Procedures
7g. provide guidance to users on the access options and their implications	Procedures
8. Repository long-term preservation (The repository should …)	
8a. support long term preservation of data and metadata	System
8b. make use of sustainable software systems	System
8c. implement policies for preservation of data	Procedures

For example, to support ‘Guidelines for data upload and storage’, the requirements for the repository could include:

a) being able to support a wide variety of file and metadata types,
b) providing easy to use mechanisms for the upload of files, including technical instructions,
c) providing rules and guidelines for data upload and storage (e.g. which formats or metadata schema to use and when).

a), and b) are mainly aspects of the repository system and its technical features, whilst c) is more a function of the repository’s policies and procedures.

In the context of this study it is important to stress that only the requirements labelled as ‘system’ attributes in Table 1 were evaluated (19 of 29, or 66%). Each of these system features was assessed and its level of fulfilment within DSpace classified as:

- demonstrated
- partially demonstrated
- not demonstrated

The assessment of the requirements was performed by BT and based on publicly available information about DSpace (web pages, user manuals, Q&A pages, reports, etc.). DSpace was not contacted directly and but there was contact with the DSpace community. The Coordination Centre for Clinical Trials in Düsseldorf participated at a meeting of the German user community.

## Results

The results are summarised in this section and in Table 2.

**Table 2.** Summary of the assessment of quality criteria and the requirements.

Requirement	Result	Comment
1a. The repository should support a range of file types and metadata schema	**Demonstrated**	Flexible approach to file storage
1b. The repository should provide mechanisms for the upload of files, including instructions	**Demonstrated**	Variety of tools available, along with detailed technical guidance
2a. The repository should be able to provide links to de-identification tools and requirements	**Partially demonstrated**	Links can be established but have to be set up by system administrators
2b. The repository should implement de- identification tools	**Not demonstrated**	Not currently possible
3a. The repository should support quality control in its workflow	**Partially demonstrated**	Some quality features available, e.g. a submission review workflow, but not a full quality control system
4a. The repository should incorporate a data transfer agreement in system workflow	**Partially demonstrated**	Confirmation of a signed data transfer protocol can be required from the user, but there is no support for constructing or editing such a document
5a. The repository should use a consistent metadata schema to describe its content	**Demonstrated**	Impressive range of metadata schemes
5b. The repository should allow a customised metadata schema to be applied	**Demonstrated**	A specific metadata schema for clinical research could be implemented
5c. The repository should provide tools to help data generators to complete metadata fields	**Demonstrated**	Range of tools available
5d. The repository should make metadata openly (public) available	**Demonstrated**	Metadata are public
6a. The repository should be able to apply a primary persistent identifier system	**Demonstrated**	Use of CNRI Handle System
6b. The repository should be able to use other persistent identifiers as appropriate	**Demonstrated**	Use of other identifiers allowed (e.g. DOI)
7a. The repository should allow open access to material, with an optional embargo period	**Demonstrated**	Sophisticated embargo management as well as full open access.
7b. The repository should allow open access after web-based self-attestation of the user	**Not demonstrated**	Not currently possible
7c. The repository should offer managed access through group membership	**Demonstrated**	Functionality access through group membership (priviledged users)
7d. The repository should offer managed access through application on a case by case basis	**Demonstrated**	Possible with the request a copy functionality, but could be extended further
7e. The repository should support granular access to different parts of datasets collections	**Demonstrated**	Permissions can be assigned to a priviledged user at the item, community and collection level
8a. The repository should support long term preservation of data and metadata	**Demonstrated**	Demonstrated as far as it is a technical issue
8b. The repository should make use of sustainable software systems	**Demonstrated**	Long-term availability and maintenance of system expected

**Table 3.** Comparison between the Banzi quality criteria ^2^ and the other approaches. Grey: not considered by the Banzi criteria ^2^.

Criterion (Banzi *et al.,* 2019) ^2^	ICSU World Data System (2016) ^9^	Burton *et al.* (2015) ^8^	Science Europe (2018) ^10^	Hrynaszkiewicz *et al.* (2016) ^7^
Guidelines for data upload and storage	R9: The repository applies documented processes and procedures in managing archival storage of the data, R12: Archiving takes place according to defined workflows from ingest to dissemination			
De-identification practices before upload	R.6: The repository adopts mechanisms to secure ongoing expert guidance and feedback (either inhouse, or external, including scientific guidance, if relevant), which could also cover requirements or guidelines related to de-identification of uploaded data.			
Control of quality of data	R11: The repository has appropriate expertise to address data and metadata quality and ensures that sufficient information is available for end users to make quality-related evaluations).	C6: Quality assurance and control C7: Curation and archiving)		
Formal contract regarding upload and storage	R2: The repository maintains all applicable licences covering data access and use and monitors compliance; including conditions of use),	C4: Transparent and accountable; all policies and written agreements underpinning a repository’s processes for data management (including any legal contracts) should be properly documented	3. Data access and usage licenses; provide information about licensing and permissions	Implement data use agreements (DUAs).
Application of a metadata schema to describe contents	R8: The repository accepts data and metadata based on defined criteria to ensure relevance and understandability for data users	C5: Data and metadata fidelity	2. Metadata; use metadata standards that are broadly accepted (by the scientific community)	
Application of an identifier			1. Provision of persistent and unique identifiers (PIDS)).	Provide stable identifiers for metadata about non- public dataset(s))
Flexibility of access				
Repository long-term preservation	R3: The repository has a continuity plan to ensure ongoing access and preservation of its holdings R10: The repository assumes responsibility for long-term preservation and manages this function in a planned and documented way	C8: Reliable availability including backup C10: Preserve confidentiality, integrity and availability of the repository).	4. Preservation; ensure persistence of metadata and data,	
Transparency and accountability		C4: Transparent and accountable		Implement a transparent system for requesting access to data and reviewing requests to access data
Timely management				Allowing access to data in a timely manner and including a proportionate review of the scientific rationale, without introducing unnecessary barriers
Metadata repository			2. Enabling referencing to related relevant information, such as other data and publications and asks	
Data versioning			1. Support of data versioning	
Audit of repositories		C9: Effective audits		
Adequate funding and staff	R5: The repository has adequate funding and sufficient members of qualified staff managed through a clear system of governance to effectively carry out the mission			
Disvoverability	R13: The repository enables users to discover the data and refer to them in a persistent way through proper citation			
Technical infrastructure	R15: The repository functions on well-supported operating systems and other core infrastructural software and is using hardware and software technological appropriate to the services it provides to its designated community. R16. The technical infrastructure of the repository should provide for protection of the facility and its data, product, services, and users			
Authenticity and integrity of the data	R7: The repository guarantees the integrity and authenticity of the data	C10: Preserve confidentiality, integrity and availability of the repository	Enable data authenticity and integrity	

### Guidelines for data upload and storage

DSpace exhibits a flexible approach to file storage by supporting a range of file types and metadata schemas (1a demonstrated). With a variety of tools available, along with detailed technical guidance, it also provides mechanisms for upload of files, including instructions (1b demonstrated).

### De-identification practices before upload

The DSpace system has no published requirements or guidelines relating to the de-identification of uploaded data. It is the submitter’s responsibility to ensure that documents are consistent with current standards, guidelines and policies from official bodies and scientific organisations. The submitter is, however, able to use links to requirements, guidelines and/or tools, if these are established by the system’s administrator (2a partially demonstrated). As far as we could tell, neither the DSpace repository system nor the user community have implemented de-identification tools or programs, able to perform and document de-identification on an existing dataset (2b not demonstrated). Having said that it is worth noting that, should such support tools be created, DSpace does provide a task management system (known as the 'Curation System') in which such tools can be integrated and configured.

### Control of quality of data

The control of the quality of data is more a question of procedures and workflow around a repository than technical features available in a particular system. Nevertheless, there are some technical features that could facilitate a quality control workflow. Some of these features are available within DSpace, usually as optional and configurable additions to the data upload process but they are limited to a predefined review workflow. This covers a single reviewer workflow, collection’s workflow steps and a score review workflow. This is certainly an important feature but does not correspond to a full quality-controlled process, which needs additional features like monitoring and tracking uploads, rejections, edits; reports about reviews in process and performed, etc. (3a partially demonstrated).

### Formal contract regarding upload and storage

A formal data transfer contract signed by the data generator and the repository administrator should be a prerequisite for transferring clinical trial data to a repository, not least to clarify potential legal responsibilities under data protection legislation. At the end of the manual submission process in DSpace, the submitter (data generator) is asked to grant the repository service an appropriate distribution license (different licences can be made available to different user communities). The distribution license can be edited or customised, however, the platform does not provide a user interface to do this easily. Agreeing a distribution licence is not the same, however, as enforcing a data transfer agreement. Confirmation of the existence of a signed data transfer protocol can be required from the user, i.e. integrated within the distribution licence, if implemented. The demonstrator repository is not, however, able to provide support for constructing and editing such a document (4a partially demonstrated).

### Application of a metadata schema to describe contents

DSpace can support multiple extended metadata schemas for describing an item. A qualified Dublin Core metadata is provided by default. Multiple schemas can be configured, and metadata fields selected from a mix of configured schemas to describe items (5a demonstrated). In addition, a new metadata schema can be created. In the demonstrator repository, the ECRIN Clinical Trial Metadata Schema was created ^11^ (5b demonstrated). DSpace has several tools to help data generators export content and metadata, ingest content and metadata tools and batch edit metadata (5c demonstrated). DSpace offers OAI-MPH, a standard protocol for metadata harvesting. Metadata are public in DSpace. Communities, Collections and Items are discoverable in the browse and search systems regardless of read authorisation. Therefore, everyone can access metadata of items openly (5d demonstrated).

### Application of an identifier

DSpace uses the CNRI Handle System primarily to create a persistent identifier for every object (item, collection and community) stored in the system (6a demonstrated). DSpace also allows other persistent identifiers, such as a digital object identifier (DOI), to be applied to data sets to improve discoverability and to allow correct citation in DSpace. This is in parallel to the Handle System (6b demonstrated).

### Flexibility of access

DSpace has sophisticated embargo management as well as full open access. Embargo settings allow submitters to define embargoes linked to specific dates, that by default are applied to all anonymous (non-administrator) access requests. Advanced embargo settings can be used to apply (or exclude) embargo policies for particular user groups (7a demonstrated). The DSpace system supports several common authentication systems, but web based self-attestation is not supported (7b not demonstrated). In this context the term 'self-attestation' refers to a registration like process where the user first has to provide information about themselves, including their contact details, and give details of the purpose for which they intend to use the data, together with any other information required by the data managers. Email details would then normally be verified (by clicking on a validation link sent to the address provided) before access would be granted.

Resources can be made available only to certain "privileged" users, and this functionality allows access through group membership to be implemented (7c demonstrated). The ‘request a copy’ functionality exists in DSpace to facilitate access in cases when uploaded content is not openly shared. With this feature, the data submitter or owner interacts directly with the requester on a case-by-case basis. More complex request evaluation processes, for example involving a data access committee, are not directly supported in DSpace, though could in theory be integrated into any dialog between the requestor and the data submitter (7d demonstrated). The DSpace administrator can assign permissions to a privileged user at the item, community and collections level, allowing granular access to different parts of datasets collections (7e demonstrated).

### Long-term preservation and sustainability

These are two related issues, one dealing with the preservation of the data in the long term, the second with the sustainability of the repository itself. A repository’s longevity will mainly be dependent on resourcing and institutional commitment, and given the inevitable uncertainties around both of these a clear policy about what should happen to data if a repository is closed would clearly be a requirement for most potential users., At a technical level, however, provides some support for long term preservation mechanisms, e.g. checksums can be applied and verified on all items. It can also be integrated with the open source archiving system Archivematica, allowing the generation of system-independent Archival Information Packages (AIPs) ^12^ (8a, in so far it is a technical issue, demonstrated). DSpace also claims to have implemented a strategic plan for sustainability. Because it uses open technology, has a broad dissemination and usage, with a large user community and many diverse applications, the long-term availability and maintenance of the system is expected, if not guaranteed (8b demonstrated).

## Discussion

### Assessment of the demonstrator repository

The performance in supporting sensitive personal data such as that from clinical trials was strong, with 14 requirements demonstrated (74%). This included strong support for different file types and metadata systems, a range of access control systems, including embargoes and granular access management, an integrated persistent identifier scheme plus support for other identifiers like DOIs, and good support for data management in the long-term.

Of the two areas that were not demonstrated at all, the first – the inability to incorporate de-identification tools in the submission workflow – is arguably an over ambitious requirement. Although general techniques certainly exist for de-identification this should normally be an exercise that is planned, documented and tested on a study-by-study basis, rather than an automatic process. Having links available to de-identification resources is probably a more realistic requirement.

The second missing requirement, the lack of a self-attestation system, is a feature that some data generators might want to use, as it requires much less administrative overhead then setting up access rights for groups and individuals. It would require an administrator to define the fields required for self-attestation and, like the current user registration process, it could be backed up by a system requiring confirmation of the email address given. Given the range of other access options available in DSpace it may not be a serious omission, but it is a missing feature that would be ‘nice to have’.

Of the three areas that were partially demonstrated, the need for repository managers to establish links to de-identification and other tools, rather than have them built-in to the system, may represent an additional task but it is one that should be relatively easy to do. It can also be argued that this approach is more flexible, and easier to keep up to date, than a set of links integrated into the system.

The second partially demonstrated area related to quality control. The submission workflow allows for up to three review stages, which is good, but few other elements of quality control and monitoring seemed to be built into the system. For repository managers handling sensitive data, it would be useful to have reports on upload and access or access request activity, and the ability to integrate checklists of required features or information (such as de-identification status, metadata completeness, access types allowed or identifiers applied), as might be applied during the review process, to tag on the data itself (i.e. within internal system metadata). This would allow the status of the data in the repository to be better monitored and potential issues with data quality and/or legal issues to be more quickly identified.

The third partially demonstrated issue related to data transfer agreements, governing the terms of data upload and storage. Sensitive data requires more than a simple upload to a repository because, unless the data is fully anonymised, there are likely to be legal issues that need to be clarified, for instance exactly which institution is acting as the Data Controller, as that term is defined in the General Data Protection Regulation (GDPR). (At the very least, the legal status of the data needs to be clear, i.e. does it fall under data protection legislation, and if so which, or is it exempt from such consideration because of the way it has been prepared.) In addition, there may be questions about who is responsible for versioning data if it is changed, for paying any associated costs, about the access management required, and who needs to review access requests if access is managed (etc.). These considerations go well beyond any general agreement whereby data generators simply grant the repository the right to make their data available under a selected licence – and for sensitive data they may need to be considered on a study by study basis.

It would therefore be very useful if – as a configured option – the system could enforce a clear check that such a data transfer agreement was in place, preferably with the date of its application. (At the moment that seems possible, but a rather complex workaround is required.) It would be even better if the system could also indicate where the data transfer agreement was stored and link to it, or even display its provisions within the system. Ideally, a mature system would even allow the agreement to be drafted and agreed within the system, as part of a private interchange between the data uploader and the repository.

### Weaknesses of the study

A limitation of the study is that it is focusing only on the 8 repository features defined in Banzi *et al.* ^2^. Other quality features not considered here may also be very important, for example good data security. This study should therefore be seen as a starting point, which will need further extension, perhaps using alternate approaches and systems (see next section).

We focused on attributes that we thought were particularly important for clinical trial and similar data. Aspects of quality for data repositories that have been cited by other authors, but which have not been explicitly considered in our approach include:

- Transparency and accountability
- Timely management
- Metadata repository
- Data versioning
- Auditing of repositories
- Adequate funding and staff
- Discoverability
- Technical infrastructure

Transparency and accountability have been referenced by Hrynaszkiewicz *et al.* ^7^ and by Burton *et al.* ^8^. Allowing access to data in a timely manner and including a proportionate review of the scientific rationale, without introducing unnecessary barriers has been formulated by 7. Science Europe supports the idea of a metadata repository, enabling referencing to related relevant information, such as other data and publications and asks for support of data versioning ^10^. Effective audits are proposed by Burton *et al.* ^8^. The ICSU World Data System requires that the repository has adequate funding and sufficient members of qualified staff managed through a clear system of governance to effectively carry out the mission and that the repository enables users to discover the data and refer to them in a persistent way through proper citation ^9^. The ICSU World Data System ^9^ requires that the repository functions on well-supported operating systems and other core infrastructural software and is using hardware and software technological appropriate to the services it provides to its designated community. In addition, the technical infrastructure of the repository should provide for protection of the facility and its data, product, services, and users ^8^. The need to try and integrate these different approaches to assessing data repositories is discussed in the next section.

Another weakness of the study is that the assessment of the quality criteria is (necessarily) subjective – the criteria are not quantitative. In our approach, a rather simple scale based upon “demonstrated”, “partially demonstrated” and “not demonstrated” was used. The definition of the different categories may not have been precise enough to give an accurate representation of the repository’s functioning.

Finally, there may be an issue related to the sources and completeness of the information used. We only took publicly available information about DSpace into consideration (web pages, user manuals, Q&A pages, reports, etc.). We did not contact DSpace directly and were not in contact with their developers. We did, however, participate at a meeting of the German user community and had discussions with a DSpace user. It should be noted, however, that transparency has been formulated as one the main principles for trusted repositories: “In order to select the most appropriate repository for a particular use case, all potential users benefit from being able to easily find and access information on the scope, target user community, policies, and capabilities of the data repository.” ^13^. As a consequence, publicly available information should be sufficient to basically assess a repository.

### Approaches and systems for assessing the quality of repositories

There are overarching general principles that address aspects around data management and data repositories on a very high level. In the FAIR principles, it is formulated that data should be Findable, Accessible, Interoperable and Reusable ^14^. The TRUST principles formulate guidance for digital repositories of research data with a focus on Transparency, Responsibility, User focus, Sustainability and Technology ^13^. Concrete guidelines, recommendations and best practice for data sharing and for trusted repositories should follow these principles and should provide concrete help for implementation of these principles.

Different approaches have been used to assess the quality of repositories dedicated to data sharing, both of sensitive data and more generally, with different emphases laid upon different features. For instance, Hrynaszkiewicz *at al*. ^7^ proposed additional features for data repositories to better accommodate non-public clinical datasets, including Data Use Agreements, whilst Burton *et al*. ^8^ introduced the term “Data Safe Haven”, for sensitive data, and provided 12 criteria that characterised such a haven.

The Core Trustworthy Data Repositories Requirements ^9^ are intended to reflect the characteristics of trustworthy repositories (for all types of data). All requirements are mandatory and are equally weighted, standalone items. Although some overlap is unavoidable, duplication of the evidence sought among requirements has been kept to a minimum where possible. The choices contained in the supplied checklists (e.g., repository type and curation level) are not considered to be comprehensive, and additional space is provided in all cases for the applicant to add ‘other’ (more idiosyncratic) information. This and any comments given may then be used to refine such lists in the future. The CoreTrustSeal Board offers all interested data repositories a core-level certification based on the DSA–WDS Core Trustworthy Data Repositories Requirements catalogue and procedures ^9^.

One initiative of Science Europe ^10^ was to develop a set of core requirements for data management plans (DMPs), as well as a list of criteria for the selection of trustworthy repositories where researchers can store their data for sharing. The different approaches are compared in Table 3. In light of the development of the European Open Science Cloud (EOSC) and the increasing pressure for data sharing, these requirements and criteria should help to harmonise rules on data management throughout Europe. This will aid researchers in complying with research data management requirements even when working with different research funders and research organisations.

In general, it may be necessary to better distinguish between criteria that are properties of the underlying infrastructure (e.g. staff preparation, physical security, logical security, appropriate technology) and those which are more tightly coupled to a specific repository system. In fact, we would suggest that there are three (overlapping) ‘layers’ of attributes that need to be considered – those associated with the underlying organisational infrastructure, those linked to the repository’s technical systems, and those derived from procedures and workflows *.* Future attempts to assess the quality of repositories should perhaps consider these layers more explicitly. In this study we were focused on the ‘system’ attributes, but a broader description and assessment of a demonstrator repository should examine all three aspects, perhaps across each of the three main functional areas of a data repository, i.e. data upload, data storage and data access.

None of the approaches described above is sufficient to classify the quality of repositories for clinical trial data, as pointed out by Banzi *et al*. ^2^. It may be that we need to differentiate criteria that should apply to all or most data repositories from those that only apply, or become more significant, in the context of particular types of data, like IPD. A general assessment, and especially a general ‘score’, of repositories may therefore be less meaningful than an assessment for particular types of data or data usage. Despite these difficulties we believe that it would be useful to try and achieve a consensus about what ‘quality’ means in terms of data repositories, in different contexts, both to support repository managers and to help guide and promote their use by researchers.

## Conclusion

We assessed the suitability of DSpace to support a repository of sensitive data, such as that from clinical trials, using quality criteria that we had previously identified as being critical to managing such data. Technically, the system was able to support most of the features required, including the necessary support for metadata and identifiers, though there are areas – for instance explicit support of data transfer agreements – where support could be improved. Of course, in a productive repository, appropriate policies and procedures would be needed to direct the use of the available technical features. A technical evaluation should therefore be seen as indicating a system’s potential, rather than being a definite assessment of its suitability. DSpace clearly has considerable potential in this context and appears a suitable base for further exploration of the issues around storing sensitive data.

This work should stimulate the discussion about quality assessment and certification of repositories. The discussion is of particular importance for repository managers as well as standardising organisations in the field (e.g. Data Seal of approval). Another target group are researchers willing to deposit data in a repository, who have an interest that definite quality criteria are fulfilled by the repository.

## Data availability

All data underlying the results are available as part of the article and no additional source data are required.

The ECRIN demonstrator repository for clinical trial data: http://90.147.75.211:8080/xmlui/

Additional information on the CORBEL project is available on the CORBEL website ( https://www.corbel-project.eu/home.html).
