# Supplementary material for: Assessment of a demonstrator repository for individual clinical trial data built upon DSpace
Source: F1000Res. 2020 Jun 25;9:311. Originally published 2020 Apr 29. [Version 2] doi: 10.12688/f1000research.23468.2 (PMC7268148; doi:10.12688/f1000research.23468.2)
Supplement: Peer review history

# Peer review: Reviewer response for version 2

**DOI:** 10.5256/f1000research.27604.r65528

**Published:** 2020-06-26

**Authors:** Agustina Martinez-Garcia

**Affiliations:** 1 University of Cambridge, Cambridge, UK

No further comments: the authors have addressed suggestions and amendments in this version.

 There is only a minor typo: the sentence "A formal comparison between the systems" in the Methods section appears twice.

Is the work clearly and accurately presented and does it cite the current literature?

Yes

If applicable, is the statistical analysis and its interpretation appropriate?

Not applicable

Are all the source data underlying the results available to ensure full reproducibility?

Yes

Is the study design appropriate and is the work technically sound?

Partly

Are the conclusions drawn adequately supported by the results?

Yes

Are sufficient details of methods and analysis provided to allow replication by others?

Yes

Reviewer Expertise:

Digital archiving, digital repository platforms, research data management

I confirm that I have read this submission and believe that I have an appropriate level of expertise to confirm that it is of an acceptable scientific standard.

# Peer review: Reviewer response for version 1

**DOI:** 10.5256/f1000research.25899.r63998

**Published:** 2020-06-02

**Authors:** Rob Baxter

**Affiliations:** 1 EPCC, University of Edinburgh, Edinburgh, UK

The article describes a useful experiment in trialling DSpace as a candidate repository for potentially sensitive clinical trial data. The assessment criteria used focussed on the “system” level, keeping the scope manageable, and map well onto more formal existing frameworks. The conclusions that DSpace is not a bad place to start — necessary but insufficient — are sound and offer a useful guide to people faced with similar challenges in enabling the sharing of sensitive data. I have a few specific observations around methods and analysis, noted below.

 While geared more towards open data, the FAIR principles ( https://www.force11.org/fairprinciples) are an increasingly important set of criteria for research data repos and complement some of the approaches in Table 3. Perhaps they could be added to the mapping?

 There is no mention of encryption in the 8 assessment criteria, but encryption is hinted at in the software config ("PostgreSQL 9.5 (with pgcrypto installed) as the relational database back end”). For a repo system handling sensitive data, I’d like to see encryption at rest and encryption in flight as two additional criteria. Perhaps this is implicit in the experiment (the pgcrypto extension offers a tantalising hint!), and if so it’s worth making it explicit. If encryption *wasn’t* considered as a criterion, it’s worth adding an explanation: certainly encrypting archive data is controversial — what if you lose the keys? — but an Internet-accessible database of sensitive data is a worrying thing to have exposed unencrypted.

 General, automatic de-identification of data is hard, as I’m sure the authors are fully aware! While they do cover de-identification support (or rather, the lack of it) in DSpace, I wonder if they would like to comment on whether they would regard some form of basic personally-identifiable data quality checking as a “must” for repository systems dealing with sensitive data? (Looking for names, addresses, email addresses, etc. in submissions.) How easy would it be for an absent-minded researcher to upload PII into DSpace and make it publicly readable by default? Should the assessment criteria be tighter here? Perhaps this is food for future work.

Is the work clearly and accurately presented and does it cite the current literature?

Yes

If applicable, is the statistical analysis and its interpretation appropriate?

Not applicable

Are all the source data underlying the results available to ensure full reproducibility?

Yes

Is the study design appropriate and is the work technically sound?

Yes

Are the conclusions drawn adequately supported by the results?

Yes

Are sufficient details of methods and analysis provided to allow replication by others?

Partly

Reviewer Expertise:

Development & provision of large-scale data services for both open and sensitive data (the Edinburgh International Data Facility, the Scottish National Safe Haven).

I confirm that I have read this submission and believe that I have an appropriate level of expertise to confirm that it is of an acceptable scientific standard.

# Response

**Published:** 2020-06-22

**Authors:** Christian Ohmann

**Affiliations:** ECRIN, Germany

A reference to the FAIR and TRUST principles was included in the paper.

It was explained why security features end encryption were not considered in detail in the paper. 

The recommendation about exploring basic personally-identifiable data quality checking as a "must" for repository systems will be followed up in future work.

# Peer review: Reviewer response for version 1

**DOI:** 10.5256/f1000research.25899.r62923

**Published:** 2020-05-20

**Authors:** Agustina Martinez-Garcia

**Affiliations:** 1 University of Cambridge, Cambridge, UK

**Summary**

 This is the review of the research paper “Assessment of a demonstrator repository for individual clinical trial data built upon the DSpace open source platform”. The paper describes the assessment and implementation of a repository demonstrator, for the storage and dissemination of clinical trial data, with a particular focus on Individual Participant Data (IPD). The developed demonstrator is built upon the open source and community developed DSpace repository platform ( https://duraspace.org/dspace/). This repository platform is data agnostic and can be used to both serve fully open content and content that requires some form of managed access. This paper will be very useful to those looking at evaluating repository platforms for archiving and disseminating research data more generally.

 The paper focuses on describing the technical criteria used for assessing the suitability of this platform for the storage and dissemination of clinical data, although a good overview of other operational aspects such as the development of guidelines, data deposition rules and quality review in the context of repository submission workflows, is also described. It also includes a summary of the technical requirements (software dependencies and deployment infrastructure) which can be useful to others evaluating the use of this repository platform for the storage and dissemination of research data.

**Research methodology**

 Overall, the paper includes sufficient details about the methods and analysis undertaken. The authors have explored recent studies in the area, i.e. the suitability assessment presented builds upon a previous study looking at a range of existing repository platforms for sharing clinical trial data, and sensitive data more broadly. The results from this study are the basis for selecting the DSpace platform. In this respect, and although the authors include references to materials where the rationale for selecting this platform is presented, it would have been useful to include a summary table outlining key criteria and some details of the other platforms evaluated. The paper only mentions other platforms (e.g. Figshare, or Zenodo) in passing.

 One strength of the paper is that the authors reflect on and present the perceived weaknesses of their study. However, and given the sensitive nature of the data underpinning clinical trials, I found it quite surprising that data security features were not included as part of the key criteria defined for this initial assessment, given that not meeting these criteria could impact the suitability of this platform for the archival of clinical data. The authors acknowledge this weakness of their study and state that criteria relating to data security should be considered in future extensions of the study. As part of future assessment, the authors should consider looking at robust security testing of the platform, such as performing penetration testing.

 Another weakness of the study, even though the authors acknowledge it in the paper, is that they have only evaluated openly available documentation for the DSpace platform. Such documentation can often be incomplete in community-based projects, owing to potential lack of resources. More detail about why they took this approach would have been useful. Moreover, and given that DSpace is a very popular platform within the academic community as acknowledged in the paper, the authors could have informally contacted other institutions currently using the platform to find out more about their experiences of the platform when put to similar uses, and their opinion on the platform’s strengths and weaknesses.

**Content review**

 The paper reads very well, and the content structure is appropriate. The “Introduction” section sets the scene nicely and provides sufficient background information, with relevant and current literature references. One minor observation is that, when authors introduce the work of a dedicated taskforce addressing the problem of current forms of sharing clinical data, and propose to use data repositories, there is no mention of the importance of gaining consent for data archiving, sharing and re-use from research participants. This is a key barrier to data sharing, and one that we encounter as providers of Research Data Management Services, when researchers wish to deposit their data with our Institutional Repository.

 The “Methods” section is well developed: the “Technical infrastructure for the demonstrator repository” section provides useful details for those seeking to use similar platforms; and sufficient information is provided so that a similar assessment can be performed on other platforms, or for study replication (even though the analysis is partially qualitative). As mentioned earlier, it would have been useful to include a summary table outlining key criteria and some details of the other platforms evaluated for completeness.

 In the “Assessment of quality criteria for the reference implementation”, the paragraph beginning with “To promote interoperability …” is a bit unclear and contradictory. It mentions the importance of using metadata standards for describing, structuring and formatting content, which I agree is very important; but they have excluded them as part of the assessment criteria. In particular, the sentence “Here we focus on standards for metadata” is very confusing as the examples given earlier all refer to metadata standards. Is the sentence intended to mean that the study is only concerned with metadata standards and does not consider data format standards?

 The “Results” section reads very well and is clear. The summary table together with the different criteria-based subsections include relevant, high-level information about the technical assessment that has been performed. With respect to requirement 2a around de-identification tools, perhaps it is worth mentioning that, although not specifically implemented by the community, the DSpace platform does have a mechanism / framework in place (i.e. curation system) that allows for easy integration of such tools within DSpace’s standard submission workflows (see https://wiki.lyrasis.org/display/DSDOC6x/Curation+System).

 It is mentioned in the section “Formal contract regarding upload and storage” that the implemented demonstrator does not provide support for constructing and editing the distribution licence. However, the distribution licence text can be edited or customised, as we have done so in our Institutional DSpace repository instance. Perhaps, what the authors mean instead is that the platform does not provide a user interface to do this easily.

 The section about repository long-term preservation could have incorporated more detailed information about the DSpace’s platform’s capabilities around content preservation and relevant references and links to relevant literature. For example, open source integrations of the DSpace platform with preservation systems exist, e.g. integration with Archivematica ( https://figshare.com/articles/Automating_OAIS_compliant_digital_preservation_using_Archivematica_and_DSpace/11274143/1). The authors seem to mix the platform’s long-term availability based on a number of aspects such as technology sustainability plans, or wide use, with the platform’s capabilities for preservation of the repository content itself. The former is not directly related to preservation but to the long-term sustainability of the platform.

 Lastly, a number of sections in the paper talk about self-attestation functions in the context of access to repository content (requirement 7b – web-based self-attestation of the user). I am not familiar with this term, and the general reader would benefit with a clearer definition of the term and such functions. I can only guess, based on context and my knowledge of repository platforms, that the authors mean the repository’s ability for user self-registration to be able to access repository content, or functions for only giving access to content once certain information about the user has been collected and verified. E.g. the repository allows to incorporate a form asking content requesters to supply information about what uses they will make of the data, purpose of their research, contact information and /or email address to be verified, etc. If this is the case, this should be made much more explicit in the paper.

**Minor edits and structure comments**

 In the “Results” subsection of the abstract, the sentence “Two requirements could not be demonstrated (inability to incorporate de-identification tools in the submission workflow, lack of a self-attestation system) …” is not clear. It needs to be rephrased, e.g. “ability to incorporate …” and “support for self-attestation …”. Otherwise it reads as though the things in parenthesis are actually the requirements.

 In the “Conclusions” subsection of the abstract, “productive repository” should read “production ready repository” or similar.

 In the “Introduction” section, first sentence, “evironment” should read “environment.

 Table 3, third row “Control of quality of data”, C6 should read “Quality assurance” instead of “insurance”. Also, Table 3 appears much earlier (page 7) than its reference within the paper (page 11). I found this quite confusing when reading the paper as it appeared straight after Table 2, and completely out of context. It would be much clearer if the table was moved closer to its reference in the text, towards the end of the paper.

Is the work clearly and accurately presented and does it cite the current literature?

Yes

If applicable, is the statistical analysis and its interpretation appropriate?

Not applicable

Are all the source data underlying the results available to ensure full reproducibility?

Yes

Is the study design appropriate and is the work technically sound?

Partly

Are the conclusions drawn adequately supported by the results?

Yes

Are sufficient details of methods and analysis provided to allow replication by others?

Yes

Reviewer Expertise:

Digital archiving, digital repository platforms, research data management

I confirm that I have read this submission and believe that I have an appropriate level of expertise to confirm that it is of an acceptable scientific standard.

# Response

**Published:** 2020-06-22

**Authors:** Christian Ohmann

**Affiliations:** ECRIN, Germany

The process of selection of DSpace as platfom for the demonstrator repository has been better explained. A table outlining key criteria of other platforms was not included but the information can be extracted from a reference.given.

 The reasons why missing security features and encryption were not considered in the paper were explained.

 A statement was included to motivate the use mainly public information.

 The importance of consent was stressed in the introduction.

 .

 The confusion over metadata was clarified.

 In the section « De-identification practices », a line was added in response to the reviewers comment.

 In « Formal contract regarding upload and storage » an explanation reflecting the comment of the reviewer was given.

 The section about « Long term preservation and sustainabiltiy » has been renamed and rewritten.

 In the section « Flexibility of access » the meaning of the term self-attestation has been clarified. .
